# Lignin as a Functional Green Coating on Carbon Fiber Surface to Improve Interfacial Adhesion in Carbon Fiber Reinforced Polymers

**DOI:** 10.3390/ma12010159

**Published:** 2019-01-06

**Authors:** László Szabó, Sari Imanishi, Fujie Tetsuo, Daisuke Hirose, Hisai Ueda, Takayuki Tsukegi, Kazuaki Ninomiya, Kenji Takahashi

**Affiliations:** 1 Institute of Science and Engineering, Kanazawa University, Kakuma-machi, Kanazawa 920-1192, Japan; sarin0509@stu.kanazawa-u.ac.jp (S.I.); tfujie@p.kanazawa-u.ac.jp (F.T.); dhirose@se.kanazawa-u.ac.jp (D.H.); 2 Innovative Composite Center, Kanazawa Institute of Technology, 2-2 Yatsukaho, Hakusan 924-0838, Japan; h-ueda@neptune.kanazawa-it.ac.jp (H.U.); tsukegi@neptune.kanazawa-it.ac.jp (T.T.); 3 Institute for Frontier Science Initiative, Kanazawa University, Kakuma-machi, Kanazawa 920-1192, Japan; ninomiya@se.kanazawa-u.ac.jp

**Keywords:** Carbon fiber, epoxy composite, cellulose derivative, lignin, surface modification, interfacial adhesion

## Abstract

While intensive efforts are made to prepare carbon fiber reinforced plastics from renewable sources, less emphasis is directed towards elaborating green approaches for carbon fiber surface modification to improve the interfacial adhesion in these composites. In this study, we covalently attach lignin, a renewable feedstock, to a graphitic surface for the first time. The covalent bond is established via aromatic anchoring groups with amine functions taking part in a nucleophilic displacement reaction with a tosylated lignin derivative. The successful grafting procedures were confirmed by cyclic voltammetry, X-ray photoelectron spectroscopy, and field emission scanning electron microscopy coupled with energy dispersive X-ray spectroscopy. Both fragmentation and microdroplet tests were conducted to evaluate the interfacial shear strength of lignin coated carbon fiber samples embedded in a green cellulose propionate matrix and in a commercially used epoxy resin. The microdroplet test showed ~27% and ~65% increases in interfacial shear strength for the epoxy and cellulose propionate matrix, respectively. For the epoxy matrix covalent bond, it is expected to form with lignin, while for the cellulosic matrix hydrogen bond formation might take place; furthermore, plastisizing effects are also considered. Our study opens the gates for utilizing lignin coating to improve the shear tolerance of innovative composites.

## 1. Introduction

The green energy policy not only involves the development of novel strategies for energy harvesting, but also places special emphasis on the improvement of the energy-efficiency of already existing technologies. Based on this principle, fuel-efficiency has been considered as a crucial requirement for creating a sustainable world. Therefore, legislative bodies are putting more and more challenging fuel consumption standards into force for manufacturers of the transportation sector all over the world [1,2]; these requirements can be met reasonably by including more and more lightweight materials into the structure of the vehicle [3]. As leading high-performance lightweight materials, carbon fiber reinforced polymers (CFRPs) caught, therefore, appreciable interest both in the industrial sector and in the scientific community. Based on a steady market growth with an annual growth rate above 10% since 2010 [4], it can be anticipated that CFRPs will indispensably shape our future. However, as petroleum-based energy intensive materials, carbon fiber reinforced polymers barely meet sustainability goals, and therefore, appreciable efforts are being made to fabricate green materials for future applications.

On one hand, a considerable amount of studies have been devoted to produce carbon fibers from renewable sources, such as lignin [5]. As a result, the mechanical strength of lignin-based carbon fibers (tensile strength lying between 0.6 and 1 GPa [5]) is not so far from the strength of carbon fibers available on the market (e.g., carbon fiber T300 from Toray has a tensile strength of ~3.5 GPa), and further improvement is expected in this field. On the other hand, special emphasis has been placed on changing the thermoset matrix (dominating the CFRPs market with ~70% contribution [4]) to environmentally more benign thermoplastic polymers [6]. Furthermore, cellulose-based carbon fiber reinforced polymers are also gaining attention [7,8], and advances in this field open the gates for the preparation of fully biomass-derived CFRPs.

Nevertheless, due to the relatively inert and hydrophobic nature of the carbon fiber surface, the interfacial adhesion between the matrix and the fiber in the composite is notoriously low, leading to limitations in mechanical performance that highly depends on this factor [9,10]. Therefore, many studies have been focused on the surface modification of carbon fibers, giving rise to a wide range of successful modification techniques to increase interfacial shear strength (IFSS) [9,10,11,12,13,14,15,16,17,18,19,20,21,22,23]. At the same time, most of the studies hardly take sustainability issues and green chemistry principles into account as the synthetic steps usually involve aggressive chemicals to bias the graphitic surface.

Keeping in mind green chemistry principles [24], and also inspired by recent achievements in our laboratory in respect to biomass valorization, we intended to utilize lignin as a functional green coating on carbon fiber surface to improve interfacial properties of a cellulose-based (cellulose propionate, thermoplastic), and a commercially used epoxy (thermosetting) composite. While an epoxy resin is not considered as a green matrix, we intended to include this type of polymer in our study based on advances in making epoxy resins from renewable sources [25,26,27]. Furthermore, there is an obvious need for improving the mechanical properties of these composites [9,10,28]. Cellulose propionate was chosen as a representative of green biomass-derived renewable plastics, this cellulose ester has superior characteristics over other cellulose derivatives available on the market (good processability at high temperature and high tensile strength due to its high molecular weight). Lignin is the most abundant renewable feedstock for aromatic compounds, and it is available in large quantities as a byproduct of the pulping industry [29,30]. A covalent lignin coating is expected to improve interfacial shear strength in cellulose-based composites via hydrogen bonding interactions (between free OH groups, and due to -OH----O=< interactions), and by acting as a plasticizer at the interphase [31], such a mechanical dynamical effect was shown to enhance the shear tolerance of CFRPs [32]. In an epoxy composite, covalent bond formation can be envisaged as a result of the reaction between epoxy groups of the resin and the OH groups of lignin, and plasticizing effects might also have an influence on IFSS. Our study is aimed at testing these theories by covalently binding lignin to the surface of carbon fiber and investigating the mechanical performance at the interphase.

## 2. Materials and Methods

### 2.1. Materials

Cellulose propionate (*M*_w_ ≈ 200,000 g mol^−1^ according to the manufacturer) was supplied by Scientific Polymer Products, Inc. (Ontario, NY, USA), the degree of substitution was determined to be 2.76 in our previous publication (92% of hydroxyl groups are substituted) [7,8]. The matrix has a tensile strength of ~80 MPa and a glass transition temperature (*T*_g_) of ~132 °C (Shimadzu DSC-60A Plus, Kyoto, Japan). Bisphenol type epoxy matrix was prepared using West System^®^ 105 Epoxy Resin and West System^®^ 206 Slow Hardener with a 1:5 (v/v) ratio according to the recommendations of the producer (Gougeon Brothers, Inc., Bay City, MI, USA). The epoxy matrix has a tensile strength of ~50 MPa and a glass transition temperature of ~59 °C.

Kraft lignin (low sulfonate content; *M*_w_ ≈ 10,000 g mol^−1^ according to the manufacturer; Lot # 04414PEV) was obtained from Sigma Aldrich (St. Louis, MO, USA). The OH content was determined using ^31^P-NMR measurement (JEOL 600 MHz FT-NMR spectrometer, JEOL Ltd., Tokyo, Japan) according to the procedure reported by Granata and Argyropoulos [33]. The free hydroxyl group content was calculated as follows: OH_aliphatic_ = 1.65 mmol g^−1^, OH_phenolic_ = 2.12 mmol g^−1^, OH_carboxylic_ = 0.59 mmol g^−1^, OH_total_ = 4.36 mmol g^−1^ (^31^P-NMR spectrum is shown in Figure S1).

Carbon fiber T700SC-12000-50C (~7 μm fiber diameter; ~4.9 GPa tensile strength) was purchased from Toray Industries (Tokyo, Japan). The fibers have nominally ~1% sizing agent on the surface, therefore, in order to access the graphitic surface for the grafting reactions, a cleaning procedure was applied [7,8], the removal of the commercial coating was evidenced in our previous report [7].

3-Ferrocenylpropionic anhydride, anhydrous dimethyl sulfoxide, anhydrous pyridine, and 4-[(*N*-Boc)aminomethyl]aniline were provided by Sigma-Aldrich (St. Louis, MO, USA). Acetonitrile and hydrochloric acid were from Naclai Tesque, Inc. (Kyoto, Japan). *Tert*-butyl nitrite (isoamyl nitrite), *ortho*-dichlorobenzene, 1,4-dioxane, *p*-toluenesulfonyl chloride (tosyl chloride, TsCl), 4-dimethylaminopyridine (DMAP), and tetrabutylammonium hexafluorophosphate were purchased from Tokyo Chemical Industry Co., Ltd. (Tokyo, Japan). All the other solvents were obtained from Kanto Chemical (Tokyo, Japan).

### 2.2. Methods

#### 2.2.1. Synthesizing Lignin Derivatives

**Figure 1:**
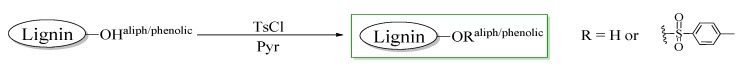
Reaction scheme for synthesizing tosylated lignin. Abbreviations: –OH^aliph^—aliphatic hydroxyl groups in lignin (1.65 mmol g^−1^ as determined by ^31^P-NMR measurement); –OH^phenolic^—phenolic hydroxyl groups in lignin (2.12 mmol g^−1^ as determined by ^31^P-NMR measurement); TsCl—tosyl chloride; and Pyr—pyridine.

One gram kraft lignin (4.36 mmol OH content) was dispersed in 100 mL pyridine. Under continuous stirring, 0.83 g tosyl chloride (~1 equivalent in respect to OH content) dissolved in 20 mL pyridine was added dropwise to the latter solution in 30 min. The reaction was conducted at 8 °C for 24 h, and the product was obtained by pouring the reaction mixture into cold ethanol. The resulting product was washed with acetone and dried in a vacuum oven at 50 °C for 3 days. The afforded tosylated lignin (Figure 1) showed good solubility in pyridine and DMSO. Since the product was not soluble in the conventional solvent system of the ^31^P-NMR measurement [33], the OH content and successful tosylation was confirmed by elemental analysis (Vario ELCUBE and Vario EL III, Elementar Analysensysteme GmbH, Langenselbold, Germany). From the elemental analysis results, we calculated that the OTs content amounts to 0.26 mmol g^−1^.

#### 2.2.2. Carbon Fiber Surface Modification

##### Grafting 4-(Aminomethyl)Benzene Functions onto the Carbon Fiber Surface

**Figure 2:**
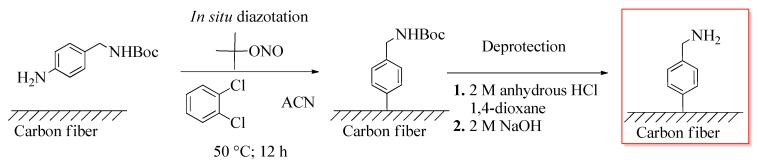
Reaction scheme for grafting 4-(aminomethyl)benzene functions onto the carbon fiber surface. Abbreviations: Boc—*tert*-butyloxycarbonyl protecting group; and ACN—acetonitrile.

4-(Aminomethyl)benzene functions were deposited on the carbon fiber surface using 4-[(*N*-Boc)aminomethyl]aniline as the starting molecule (Figure 2). The diazonium salt (29.2 μmol/mg fibers) was in situ prepared with isoamyl nitrite (58.4 μmol/mg fibers) in *ortho*-dichlorobenzene/acetonitrile (2:1 volume fraction) mixed solvent system, and decomposed by heating (50 °C, 24 h) in the presence of carbon fibers. After the reaction, the fibers were washed thoroughly with dichloromethane, deionized water, and acetone. The deprotection step involved the immersion of the fibers in 2 M anhydrous HCl solution (in 1,4-dioxane), followed by a neutralization step with 2 M aqueous NaOH solution. The functionalized fibers were washed again with dichloromethane, deionized water, and acetone, and dried in a vacuum oven at 50 °C for 24 h. This free radical-mediated grafting procedure was shown to be a benign means of functionalization not affecting key single carbon fiber mechanical parameters [17]. A detailed description of the experimental procedure is given in our previous publication [7].

##### Derivatization of 4-(Aminomethyl)Benzene Functions for Determining the Grafting Density

**Figure 3:**
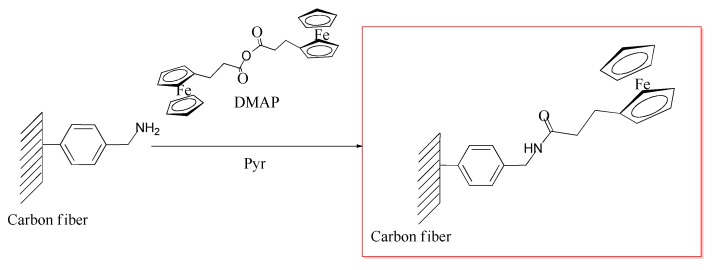
Reaction scheme for binding electrochemically active structure to the 4-(aminomethyl)benzene functions. Abbreviations: DMAP—4-dimethylaminopyridine; and Pyr—pyridine.

The deposited 4-(aminomethyl)benzene functions were further derivatized in order to be able to precisely quantify the amount of grafted structures on the surface. For this purpose, an electrochemical probe (Figure 3) was covalently linked to these structures according to the following procedure. Briefly, 25 mg functionalized fibers were immersed in 50 mL pyridine and reacted with 0.36 g 3-ferrocenylpropionic anhydride (0.73 mmol) in the presence of 0.09 g DMAP (0.73 mmol) at 80 °C for 24 h. The fibers thus obtained were washed with dichloromethane, deionized water, and acetone, and dried in a vacuum oven at 50 °C for 24 h.

##### Grafting Lignin Derivative onto the Carbon Fiber Surface

**Figure 4:**
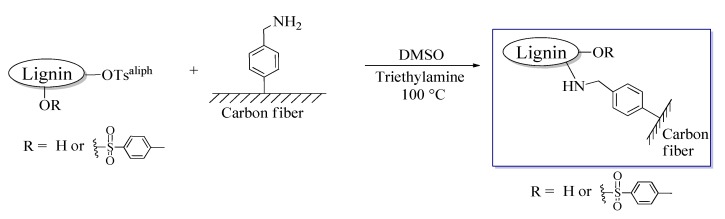
Immobilizing lignin derivative on the carbon fiber surface. Abbreviations: –OTs^aliph^—tosylated aliphatic hydroxyl groups; and DMSO—dimethyl sulfoxide.

Five gram tosylated lignin was dissolved in 50 mL DMSO and added to a solution containing 0.1 mL triethylamine (0.73 mmol) and 25 mg 4-(aminomethyl)benzene functionalized carbon fiber in 20 mL DMSO (Figure 4). The reaction was conducted at 100 °C for 24 h. The fibers were washed, as previously stated, and dried at 50 °C for 24 h in a vacuum oven.

#### 2.2.3. Mechanical Tests

##### Fragmentation Test

Fragmentation test was conducted according to our previous studies using cellulose propionate as polymer matrix [7,8]. A hydraulic hot-press machine (Type MH-10, Imoto Machinery Co., Kyoto, Japan) was used to prepare polymer films and single fiber composites (10 specimens from each type of carbon fibers). Plastic film with a thickness of ~0.15 mm was prepared by firstly placing 1 g of cellulose propionate between the clams of the hot press machine at 203 °C for 7 min, followed by pressing with 45 kN load for 3 min. The film was removed after the temperature dropped below 193 °C. Single fibers were placed between two plastic films and the same hot pressing process was applied. Thereafter, standard dumbbell specimens (the exact dimensions are given in our previous work [7]) were cut from the single fiber composite films. The specimens were elongated longitudinally with a Shimadzu Autograph AG-X Plus 5 kN tensile tester (Kyoto, Japan) applying a crosshead speed of 0.5 mm min^−1^. The fragmentation process was monitored with a high resolution digital camera (N.O.W.-D2X3Z-KSH, Nihonkouki, Aoki, Japan), and the fragment size after saturation (no shorter fragment size observed) was determined using an AUSB-K version 14.4 program (Nihonkouki, Aoki, Japan) with the help of an objective micrometer (Shibuya Optical Co., Ltd., Wako, Japan). For each specimen, the elongation at break exceeded 20 mm, and the saturation point could be clearly achieved. The Kelly-Tyson model [34,35] is applied to calculate the apparent interfacial shear strength according to Equations (1)–(3):

*τ* = (*σ*_fu_ × *d*)/(2·*l*_c_) (1)

*l*_c_ = 4/5·*l* (2)

*σ*_fu_ = *σ*_l_ × (*l*_l_/*l*_c_)^(1/*m*)^ (3)

where *σ*_fu_ is the fiber tensile strength at critical length, *d* is the fiber diameter and *l*_c_ denotes critical length, which is defined according to Equation (2) involving the average fragment size (*l*). The *σ*_fu_ can be calculated after *σ*_l_ is determined from single fiber tensile strength experiments conducted at *l*_l_ gauge length [35,36]. The Weibull modulus (*m*) represents the data spread of single fiber tensile strength experiments.

##### Microdroplet Test

Microdroplet experiment was conducted on an HM410 equipment designed for the evaluation of fiber/resin composite interface properties, manufactured by Tohei Sangyo Co., Ltd. (Tokyo, Japan). Droplets on single fibers were prepared with diameters between 80–100 μm (droplets with sizes outside this range were not measured), the experimental setup is shown in Figure S2. For preparation of droplets from the epoxy matrix, epoxy resin and hardener was mixed in a 1:5 (*v/v*) ratio according to the recommendations of the producer. Single fibers fixed on a metal frame were immersed in this mixture, and droplets were formed due to the Rayleigh instability. The samples were kept in a drying oven at 80 °C for 3 days. In case of cellulose propionate, the polymer was melted on a TJA-550 hot plate (AS ONE Corporation, Osaka, Japan) at 235 °C (5 min), and then droplets were formed at 250 °C (3 min). These samples were kept at room temperature. The microdroplet test was conducted at room temperature with a pull-out speed of 0.06 mm min^−1^ and 1 N load cell was applied. The interfacial shear strength was calculated for the maximum load (*F*) according to Equation (4).

*τ* = *F*/π*dL* (4)

where *d* is the fiber diameter and *L* is the embedded length (droplet length).

Fifty specimens were prepared for the microdroplet test from each type of carbon fibers. Significance analysis was performed using multiple *t*-test, assuming that the data represents a population with equal variance and *α* = 0.05.

#### 2.2.4. Surface Analysis

X-ray photoelectron spectroscopy (XPS) was conducted on a Thermo Scientific K-Alpha X-ray Photoelectron Spectrometer System equipped with an Al K_α_ monochromated X-ray source (1486.6 eV) having a power of 36 W (12 kV × 3 mA) (Waltham, MA, USA). The analysis spot size was set to 400 μm. The binding energy scale was calibrated using the hydrocarbon C1s peak at 285.0 eV. High-resolution C1s, O1s, N1s, and S2p spectra were recorded at 20 eV pass energy with 0.1 eV resolutions. The spectra were analyzed with a Thermo Scientific Avantage Software version 5.89 (Waltham, MA, USA). Background correction was executed using a built-in Smart algorithm and peak fitting was performed with the Powell method applying a Gauss-Lorentz Mix algorithm.

Field emission scanning electron microscopy (FE-SEM) images were obtained with a JSM-7610F system (JEOL, Tokyo, Japan), which was equipped with a JEOL EX-230**BU EX-37001 Energy Dispersive X-Ray Analyzer (JEOL, Tokyo, Japan) allowing to acquire EDX spectra and perform chemical mapping experiments. The FE-SEM analysis was conducted with a working distance of 8 mm applying an accelerating voltage of 15 kV.

SEM images were recorded on a Hitachi S4500 system (accelerating voltage of 15 kV, Hitachi, Ltd., Tokyo, Japan). Samples were coated with Au/Pd layer for 40 s using a Hitachi E1030 ion sputter (Hitachi, Ltd., Tokyo, Japan).

Cyclic voltammetry experiments were performed using an ALS/CHInstruments Electrochemical Analyzer Model 1200A potentiostat connected to an SVC3 voltammetry cell (ALS Co., Ltd., Tokyo, Japan). The three electrode system consisted of a platinum counter electrode (ALS Co., Ltd., Tokyo, Japan), an Ag/Ag^+^ non-aqueous reference electrode (RE-7, ALS Co., Ltd., Tokyo, Japan), and carbon fiber was used as working electrode attached to the terminal with a copper tape. The cell containing 0.1 M tetrabutylammonium hexafluorophosphate in acetonitrile (supporting electrolyte) was cycled between −0.4 V and 0.4 V, with a scan rate of 0.1 V s^−1^. The area under the reduction and oxidation peak was integrated to obtain the charge (in Coulomb) transferred per 1 s, and by dividing this value with the scan rate we could calculate the total charge transferred (*Q*). The surface coverage can be determined by the following equation in case of the ferrocene/ferrocenium couple (one electron transfer, *m* denotes the mass of the functionalized carbon fiber working electrode) [21]:

Surface coverage (mol mg^−1^) = *Q*/96485*m* (5)

The cyclic voltammetry experiment was conducted with three samples and both the reduction and oxidation peaks were taken into account when calculating (from the first cycles) the average surface coverage and the associated deviation.

## 3. Results and Discussion

### 3.1. Grafting Lignin onto the Carbon Fiber Surface

Lignin was bound covalently to the graphitic surface of carbon fibers according to Figure 1, Figure 2 and Figure 4. Our synthetic strategy involved in situ grafting of a 4-(aminomethyl)benzene moiety onto the surface using diazonium species (Figure 1) followed by a simple nucleophilic displacement reaction with tosylated lignin (Figure 4). The mechanism of functionalization via diazonium species can be explained in terms of free radical processes. Diazonium species can yield aryl radicals upon heating, these radicals can add to double bonds or to aromatic systems [37]. This type of functionalization for an extended aromatic graphitic system was reported first for carbon nanotubes [38], and later adopted to carbon fiber surface chemistry [17]. The further S_N_2 type alkylation of an amino group via the tosylated derivative of a biopolymer is a well-described reaction in the literature [39].

#### 3.1.1. Quantifying Grafted 4-(Aminomethyl)Benzene Functions on the Surface

The presence of a 4-(aminomethyl)benzene moiety, and thereby the validation of the first step of our synthetic strategy (Figure 1), has been proved in our previous study by XPS analysis [7]. However, the amount of grafted structures on the surface has not been quantified. In order to be able to build other, environmentally more benign synthetic paths on our methodology in the future, we intended to take this issue under scrutiny. For this purpose, the originally grafted 4-(aminomethyl)benzene structure was modified with an electrochemical probe (ferrocene/ferrocenium redox couple), and cyclic voltammetry experiment was performed. The cyclic voltammogram thus obtained is shown in the Supplementary Material (Figure S3), and from these data, the surface coverage was determined to be 2.96 ± 1.6 × 10^14^ molecules mg^−1^. The grafting density in our study is higher than that reported by Li et al. [12] (8.15 × 10^12^ molecules mg^−1^ for grafted 4-aminobenzene structures; they used different reaction conditions, e.g., isopentyl nitrite as reactant and water as solvent), and somewhat lower than that reported for electrochemical grafting of a diazonium salt (~1.2 × 10^15^ molecules mg^−1^ for grafted phenylacetylene structures) [21].

#### 3.1.2. Surface Characterization—Experimental Evidence for Lignin Coating

**Figure 5:**
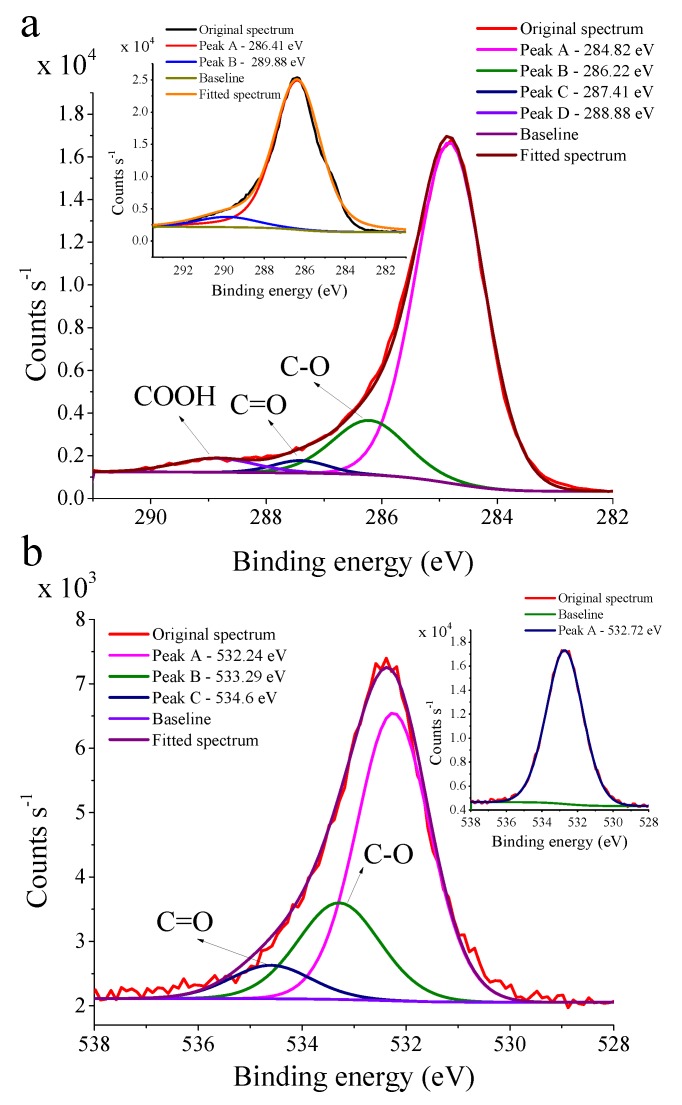
(**a**) C1s and (**b**) O1s spectra of carbon fiber sample with lignin coating, insets show the C1s (**a** Inset) and O1s (**b** Inset) spectra recorded on the clean carbon fiber surface before the functionalization procedure.

X-ray photoelectron spectroscopy experiments were performed to confirm successful functionalization of the surface with lignin, and thereby validating our synthetic procedure (Figure 1, Figure 2 and Figure 4). The high-resolution C1s and O1s spectra are shown in Figure 5a,b, respectively (survey spectrum is shown in Figure S4).

The high resolution C1s spectrum of the unfunctionalized carbon fiber surface (Figure 5a Inset) exhibits a broad and characterless peak assigned to localized (amorphous) as well as delocalized alternant hydrocarbons [40,41]. Furthermore, a broad peak arises at higher binding energies representing defect sites with –COOH functions, and π-π* shake-up peaks are also present around this region [41]. At the same time, after the functionalization procedure (Figure 1, Figure 2 and Figure 4), the high-resolution C1s spectrum (Figure 5a) indicates the presence of additional peaks that can be attributed to C–OH (286.22 eV), C=O (287.41 eV), and COOH (288.88 eV) moieties deposited on the surface [41,42]. The relatively large C–O peak suggests appreciable contribution of C–OH functions to the C1s spectrum, and therefore, an enhanced amount of these functions on the surface. Such a carbon-oxygen bond distribution profile clearly indicates the presence of lignin. To gain further knowledge about surface chemical composition, the O1s spectrum was also recorded (Figure 5b). Compared to the O1s spectrum of the unfunctionalized sample (Figure 5b Inset), the spectrum of the functionalized sample can be resolved to further components indicating C–O and C=O bonds on the surface [41], which have a similar contribution to that shown in the C1s spectrum (Figure 5a). This result additionally confirms a successful grafting procedure leading to a lignin-coated surface. In addition, the S2p spectrum of the functionalized sample (Figure S5) shows a small peak localized around the binding energies, characteristic for tosylates (~168–169 keV) [43], and the weak intensity compared to the unfunctionalized sample (Figure S5 Inset) suggests that most of the tosyl groups took place in the nucleophilic displacement reaction. We have also acquired the high resolution N1s spectra of the unfunctionalized fibers together with the 4-(aminomethyl)benzene and lignin functionalized samples (Figure S6). The N1s spectra indicate very small amounts of N on the surface of these materials. Due to the spectral similarities and very low intensities compared to the spectral noise, firm conclusions cannot be drawn from the N1s spectra.

To further analyze the bulk structure of the fibers and the near-surface layers, energy dispersive X-ray analysis was also performed (the penetration depth reaches the micrometer scale, note that the carbon fiber diameter is ~7 μm). Elemental compositions obtained from EDX analysis are shown in the Supplementary Material (Table S1). Compared to the control sample (no sizing agent, unfunctionalized), a reasonable increase in the oxygen content can be noticed for the lignin-coated sample. Furthermore, the nitrogen content remains similar to the control sample. Based on the results of the chemical mapping (Figure S7), the fiber structure does not suffer damage during our synthetic procedure, which is very important for keeping crucial carbon fiber mechanical parameters that are eventually imparted to the final composite.

The FE-SEM (Figure S8) and SEM (Figure S9) images indicate that the control fiber surface exhibits enhanced surface roughness compared to the lignin functionalized sample and the sample with a sizing agent on the surface (carbon fiber as received). The FE-SEM and SEM images point out the presence of smooth lignin coating on the surface.

### 3.2. Mechanical Tests

#### 3.2.1. Fragmentation Test

**Figure 6:**
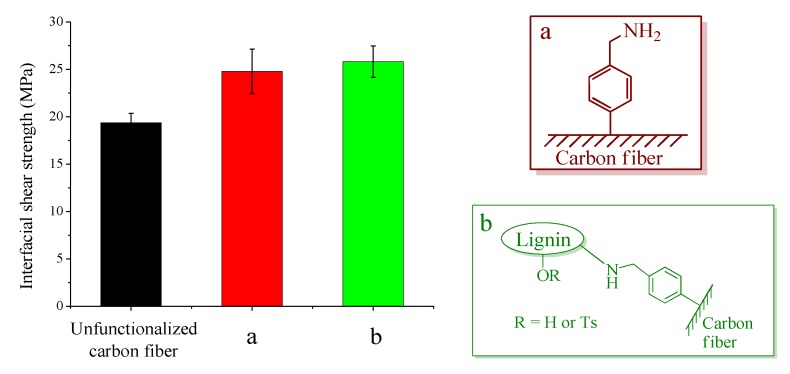
Interfacial shear strength determined indirectly using fragmentation test. The test was performed on composites prepared using cellulose propionate as matrix and unfunctionalized carbon fiber, (**a**) 4-(aminomethyl)benzene functionalized carbon fiber, or (**b**) carbon fiber containing lignin on the surface.

Dumbbell specimens for the fragmentation test were prepared using injection molding technique according to our previous studies [7,8]. As a green thermoplastic matrix, cellulose propionate was chosen for these experiments due to its superior properties among cellulose-based matrices available on the market (high molecular weight, good processability [7,8]). Epoxy resin was not used for the fragmentation test (difficulties in preparing single fiber composite for the test), but it was involved in the microdroplet experiment. The results of the fragmentation test are shown in Figure 6. The IFSS increases ~28% for 4-(aminomethyl)benzene functionalized single fiber composites compared to the unfunctionalized sample (no sizing). This phenomenon was attributed, in our previous study, to hydrogen bonding interactions between the amino groups on the surface of fibers and oxygen atoms of the cellulose propionate matrix [7]. A similarly high IFSS value was also obtained for the lignin coated carbon fiber sample. According to our theory, in this case, hydrogen bonding interactions and mechanical dynamical effects (plasticizing effect [31]) can take place, improving interfacial shear strength. The SEM images of the fracture surfaces (Figure S10) are also in line with the IFSS values since the hole surrounding the fibers after delamination is smaller as the IFSS increases, indicating stronger fiber-matrix interactions.

#### 3.2.2. Microdroplet Test

**Figure 7:**
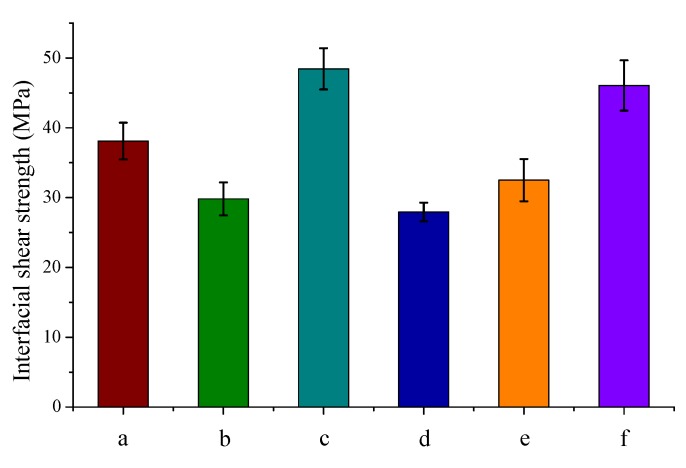
Results of the microdroplet experiments. (**a**) Epoxy resin—unfunctionalized carbon fiber (CF), (**b**) epoxy resin—4-(aminomethyl)benzene functionalized CF, (**c**) epoxy resin—lignin functionalized CF, (**d**) cellulose propionate—unfunctionalized CF, (**e**) cellulose propionate—4-(aminomethyl)benzene functionalized CF, and (**f**) cellulose propionate—lignin functionalized CF.

The microdroplet test is considered to be a direct method to evaluate the interfacial shear strength; the pull-out force is monitored in real-time during the experiment. The results of the microdroplet test are shown in Figure 7.

A decrease in IFSS is noticed for 4-(aminomethyl)benzene functionalized carbon fiber samples embedded in the epoxy matrix (compared to the unfunctionalized sample). This decrease is quite surprising since covalent bond is expected to form as a result of the reaction between the epoxy groups of the matrix and the amine functions on the surface. Such a covalent interaction between the matrix and fiber was shown to considerably increase the interfacial shear strength [20,21]. We rationalized our finding in light of the increased reactivity of 4-(aminomethyl)benzene compared to aniline (previous studies [20]) towards epoxy reagents [44]. There is a competition between surface-grafted amine functions and the amine hardener for reacting with the epoxy groups, which might lead to a less interconnected, and thus weaker, epoxy matrix near the interface. The IFSS shows appreciable increase when lignin is present on the carbon fiber surface (~27% increase compared to the unfunctionalized sample), and in this case, a covalent bond can be formed involving the hydroxyl groups of lignin and epoxy groups of the matrix.

**Figure 8:**
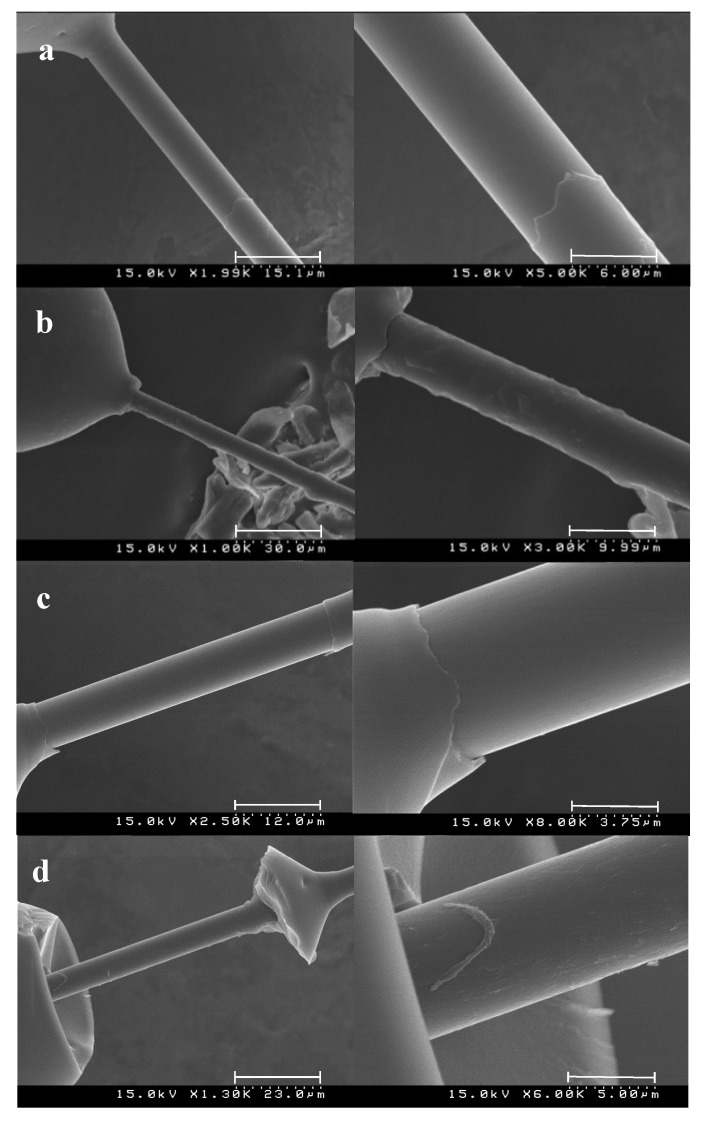
Fracture surfaces recorded after microdroplet test. (**a**) Epoxy matrix with unfunctionalized fiber, (**b**) epoxy matrix with lignin coated fiber, (**c**) cellulose propionate matrix with unfunctionalized fiber, and (**d**) cellulose propionate with lignin functionalized fiber.

When cellulose propionate is used as the matrix, the IFSS value increases as 4-(aminomethyl)benzene functions are deposited on the carbon fiber surface (~16% increase) in line with the fragmentation test (Figure 6). However, a relatively large increase in IFSS (~65% increase) is experienced for the lignin functionalized carbon fiber sample compared to the fragmentation test. A similarly large increase was obtained previously for a thermoplastic polypropylene matrix and ionic liquid sizing agent, the latter acting as a plasticizer at the interface [32]. We assume that our result cannot be only discussed in terms of hydrogen bonding interactions, but plasticizing effects might also take place. Previous reports indicate that lignin might exert a plasticizing effect for cellulose esters (glass transition temperature decreases as lignin is added to the system) [31]. The difference found between the IFSS values of the fragmentation test and the microdroplet test is attributed to the inherently different experimental approaches. While during the microdroplet test the load is placed on a small resin droplet on the fiber, during the fragmentation test, the sample is elongated using an autograph and the load is transferred to the fiber through a large amount of the matrix, in the latter process, therefore, the load transfer is less efficient. The microdroplet test should be considered to be more relevant as it is a direct measure of IFSS. In order to substantiate and visualize the results of the microdroplet test, the fracture surfaces were monitored after debonding (Figure 8).

In case of the unfunctionalized fibers (Figure 8a,c), both for the epoxy resin and for the cellulose propionate matrix, the delamination is smooth after reaching the maximum load during the microdroplet test, and furthermore, there is no remaining polymer on the surface indicating poor interfacial adhesion. When 4-(aminomethyl)benzene functions are present on the surface, however, some matrix remains on the fibers as expected (Figure S11a,b, note that the lower IFSS value for the epoxy resin is attributed to the formation of a less interconnected polymer network near the interface). In case of the epoxy resin droplet when the fibers are coated with lignin (Figure 8b), an appreciable amount of matrix remains on the surface, and matrix failure occurs indicating very strong interfacial interactions. Furthermore, for cellulose propionate matrix, a drastic failure takes place when lignin is deposited on the carbon fiber surface (Figure 8d), again the bulk matrix deteriorates before the interfacial layer and considerable amount of polymer is observable on the surface. Our results therefore indicate that, practically, the adhesion at the interface is maximized as the interfacial layer can resist more load than the matrix itself.

The effect of sizing agent on the interfacial shear strength for cellulose propionate matrix has been investigated in our previous study using the fragmentation test [7]. As the sizing agent was removed from the surface, the interfacial shear strength slightly increased (~10%). We attributed this increase to the unfavorable interactions between polar groups of the sizing agent and the hydrophilic alkyl chains of cellulose propionate. When carbon fiber is coated with lignin, an ~46% increase in interfacial shear strength can be noted compared to the fiber with sizing agent. At the same time, it appears that the presence/absence of sizing agent has only slight effect on the interfacial shear strength for an epoxy composite based on a study that used the same type of fibers and microdroplet test [45].

### 3.3. Practical Considerations

When carbon fiber surface chemistries are applied, it is crucial to avoid any damage to the fiber structure and thus keep single fiber strength. The first step of the functionalization procedure (grafting aromatic structure onto the carbon fiber surface) is known to be a nondestructive process, not impairing single fiber strength [17]. Grafting lignin onto this structure is thought to be still a benign process since there was no mechanical damage observed on the fiber.

It should be noted that we placed special emphasis on using a synthetic pathway that can be followed easily by surface analytical techniques (e.g., cyclic voltammetry, XPS spectroscopy, and FE-SEM EDX analysis). Especially because of the heterogeneity of carbon fiber surface, detecting functional groups on the surface is difficult [17,46]. Based on our results, an environmentally more benign synthetic strategy is planned to be applied and the process will be scaled-up to prepare large amount of carbon fiber reinforced composites.

## 4. Conclusions

Recent advances achieved in making carbon fibers from renewable sources, together with the successful preparation of cellulose-based carbon fiber reinforced composites, open the gates for the creation of fully bio-based high-performance composites. Nevertheless, the carbon fiber surface needs to be modified to improve the interfacial adhesion, these techniques usually involve environmentally less benign chemistries. In this study, we intended to use lignin as a functional green material to improve interfacial properties of epoxy and cellulose-based composites.

We found that covalent attachment of lignin to the carbon fiber surface leads to very strong interfacial interactions for epoxy and cellulose propionate matrices. Based on microdroplet experiments, in these composites matrixes, failure occurs before interfacial delamination could take place. Therefore, the mechanical tests suggest that covalent interactions (epoxy matrix), hydrogen bonding (cellulose propionate matrix), and plasticizing effects (cellulose propionate and epoxy matrix) might lead to improved shear tolerance materials when lignin is present on carbon fiber surface.

## Supplementary Materials

The following are available online at http://www.mdpi.com/1996-1944/12/1/159/s1. Figure S1: Quantitative ^31^P-NMR spectrum for determining OH content of kraft lignin sample (as received from Sigma Aldrich) according to Granata and Argyropoulos [31], Table S1: Elemental composition of the bulk and near-surface layer of carbon fiber samples, Figure S2: Microdroplet experimental setup, Figure S3: Cyclic voltammetry results to determine grafting density of 4-(aminomethyl)benzene structures modified with the ferrocene/ferrocenium couple (note that the cyclic voltammograms were obtained using different amount of samples), Figure S4: Survey spectrum recorded for carbon fiber sample containing lignin on the surface, Figure S5: High-resolution S2p spectra of carbon fiber sample containing lignin on the surface with inset displaying the unfunctionalized sample, Figure S6: High resolution N1s spectra of (a) unfunctionalized carbon fiber, (b) 4-(aminomethyl)benzene functionalized carbon fiber and (c) lignin functionalized samples, Figure S7: Chemical mapping experiment for carbon fiber sample containing lignin on the surface, Figure S8: FE-SEM images of (a) control carbon fiber (unfunctionalized), (b) carbon fiber with sizing agent (as received) and (c) carbon fiber containing lignin on the surface, Figure S9: SEM images of (a) control carbon fiber (unfunctionalized), (b) carbon fiber with sizing agent (as received) and (c) carbon fiber containing lignin on the surface, Figure S10: SEM images of fracture surfaces after the fragmentation test for (a) unfunctionalized carbon fiber sample, (b) carbon fiber functionalized with 4-(aminomethyl)benzene and (c) containing lignin coating on the surface, Figure S11: Fracture surfaces recorded after microdroplet test. 4-(Aminomethyl)benzene functionalized carbon fiber samples with (a) epoxy and (b) cellulose propionate matrix.

## References

[1] U.S. Environmental Protection Agency (2012). 2017 and later model year light-duty vehicle greenhouse gas emissions and corporate average fuel economy standards. Fed. Reg..

[2] Regulation of the European Parliament and of the Council (2017). Setting Emission Performance Standards for New Passenger Cars and for New Light Commercial Vehicles as Part of the Union’s Integrated Approach to Reduce CO2 Emissions from Light-Duty Vehicles and Amending Regulation (EC) No 715/2007.

[3] Kyono T., The Society of Fiber Science and Technology, Japan (2016). Life cycle assessment of carbon fiber-reinforced plastic. High-Performance and Specialty Fibers.

[4] Witten E., Sauer M., Kühnel M. (2017). Composites Market Report 2017. Market Developments, Trends, Outlook and Challenges.

[5] Fang W., Yang S., Wang X.-L., Yuan T.-Q., Sun R.-C. (2017). Manufacture and application of lignin-based carbon fibers (LCFs) and lignin-based carbon nanofibers (LCNFs). Green Chem..

[6] Yao S.-S., Jin F.-L., Rhee K.Y., Hui D., Park S.-J. (2018). Recent advances in carbon-fiber-reinforced thermoplastic composites: A review. Compos. Part B Eng..

[7] Szabó L., Imanishi S., Kawashima N., Hoshino R., Takada K., Hirose D., Tsukegi T., Ninomiya K., Takahashi K. (2018). Carbon fiber reinforced cellulose-based polymers: Intensifying interfacial adhesion between the fibre and the matrix. RSC Adv..

[8] Szabó L., Imanishi S., Kawashima N., Hoshino R., Hirose D., Tsukegi T., Ninomiya K., Takahashi K. (2018). Interphase engineering of a cellulose-based carbon fiber reinforced composite by applying click chemistry. Chem. Open.

[9] Sharma M., Gao S., Mäder E., Sharma H., Wei L.Y., Bijwe J. (2014). Carbon fiber surfaces and composite interphases. Compos. Sci. Technol..

[10] Krager-Kocsis J., Mahmood H., Pegoretti A. (2015). Recent advances in fiber/matrix interphase engineering for polymer composites. Prog. Mater. Sci..

[11] Wang Y., Meng L., Fan L., Wu G., Ma L., Zhao M., Huang Y. (2016). Carboxyl functionalization of carbon fibers via aryl diazonium reaction in molten urea to enhance interfacial shear strength. Appl. Surf. Sci..

[12] Li N., Wu Z., Huo L., Zong L., Guo Y., Wang J., Jian W. (2016). One-step functionalization of carbon fiber using in situ generated aromatic diazonium salts to enhance adhesion with PPBES resins. RSC Adv..

[13] Wang C., Chen L., Li J., Sun S., Ma L., Wu G., Zhao F., Jiang B., Huang Y. (2017). Enhancing the interfacial strength of carbon fiber reinforced epoxy composites by green grafting of poly(oxypropylene) diamines. Compos. Part A Appl. Sci. Manuf..

[14] Zho M., Meng L., Ma L., Wu G., Xie F., Ma L., Wang W., Jiang B., Huang Y. (2017). Stepwise growth of melamine-based dendrimers onto carbon fibers and the effects on interfacial properties of epoxy composites. Compos. Sci. Technol..

[15] Wu G., Ma L., Wang Y., Liu L., Huang Y. (2016). Interfacial properties and thermo-oxidative stability of carbon fiber reinforced methylphenylsilicone resin composites modified with polyhedral oligomeric silsesquioxanes in the interphase. RSC Adv..

[16] Ma L., Meng L., Wu G., Wang Y., Zhao M., Zhang C., Huang Y. (2015). Effects of bonding types of carbon fibers with branched polyethyleneimine on the interfacial microstructure and mechanical properties of carbon fiber/epoxy resin composites. Compos. Sci. Technol..

[17] Servinis L., Henderson L.C., Andrighetto L.M., Huson M.G., Gengenbach T.R., Fox B.L. (2015). A novel approach to functionalise pristine unsized carbon fibre using in situ generated diazonium species to enhance interfacial shear strength. J. Mater. Chem. A.

[18] Beggs K.M., Servinis L., Gengenbach T.R., Huson M.G., Fox B.L., Henderson L.C. (2015). A systematic study of carbon fiber surface grafting via in situ diazonium generation for improved interfacial shear strength in epoxy matrix composites. Compos. Sci. Technol..

[19] Servinis L., Gengenbach T.R., Huson M.G., Henderson L.C., Fox B.L. (2015). A novel approach to the functionalisation of pristine carbon fibre using azomethine 1,3-dipolar cycloaddition. Aust. J. Chem..

[20] Servinis L., Beggs K.M., Scheffler C., Wölfel E., Randall J.D., Gegenbach T.R., Demir B., Walsh T.R., Doeven E.H., Francis P.S. (2017). Electrochemical surface modification of carbon fibers by grafting of amine, carboxylic acid and lipophilic amide groups. Carbon.

[21] Servinis L., Beggs K.M., Gengenbach T.R., Doeven E.H., Francis P.S., Fox B.L., Pringle J.M., Pozo-Gonzalo C., Walsh T.R., Henderson L.C. (2017). Tailoring the fiber-to-matrix interface using click chemistry on carbon fibre surfaces. J. Mater. Chem. A.

[22] Eykens D.J., Stojcevski F., Hendlmeier A., Arnold C.L., Randall J.D., Perus M.D., Servinis L., Gegenbach T.R., Demir B., Walsh T.R. (2018). An efficient high-throughput grafting procedure for enhancing carbon fiber-to-matrix interactions in composites. Chem. Eng. J..

[23] Arnold C.L., Beggs K.M., Eykens D.J., Stojcevski F., Servinis L., Henderson L.C. (2018). Enhancing interfacial shear strength via surface grafting of carbon fibers using the Kolbe decarboxylation reaction. Compos. Sci. Technol..

[24] Kümmerer K. (2017). Sustainable chemistry: A future guiding principle. Angew. Chem. Int. Ed..

[25] Raquez J., Deléglise M., Lacrampe M., Krawczak P. (2010). Thermosetting (bio) materials derived from renewable resources: A critical review. Prog. Polym. Sci..

[26] Dai J., Peng Y., Teng N., Liu Y., Liu C., Shen X., Mahmud S., Zhu J., Liu X. (2018). High-performing and fire-resistant biobased epoxy resin from renewable sources. ACS Sustain. Chem. Eng..

[27] Li R.J., Gutierrez J., Chung Y.-L., Frank C.W., Billington S.L., Sattely E.S. (2018). A lignin-epoxy resin derived from biomass as an alternative to formaldehyde-based wood adhesives. Green Chem..

[28] Nazhad H.Y., Thakur V.K. (2018). Effect of morphological changes due to increasing carbon nanoparticles content on the quasi-static mechanical response of epoxy resin. Polymers.

[29] Thakur S., Govender P.P., Mamo M.A., Tamulevicius S., Mishra Y.K., Thakur V.K. (2017). Progress in lignin hydrogels and nanocomposites for water purification: Future perspectives. Vacuum.

[30] Laurichesse S., Avérous L. (2014). Chemical modifications of lignins: Towards biobased polymers. Prog. Polym. Sci..

[31] Rials T.G., Glasser W.G. (1989). Multiphase materials with lignin. VI. Effect of cellulose derivative structure on blend morphology with lignin. Wood Fiber Sci..

[32] Eykens D.J., Servinis L., Scheffler C., Wölfel E., Demir B., Walsh T.R., Henderson L.C. (2018). Synergistic interfacial effects of ionic liquids as sizing agents and surface modified carbon fibers. J. Mater. Chem. A.

[33] Granata A., Argyropoulos D.S. (1995). 2-Chloro-4,4,5,5-tetramethyl-1,3,2-dioxaphospholane, a reagent for the accurate determination of the uncondensed and condensed phenolic moieties in lignins. J. Agric. Food Chem..

[34] Kelly A., Tyson A.W. (1965). Tensile properties of fiber-reinforced metals: Copper/tungsten and copper/molybdenum. J. Mech. Phys. Solids.

[35] Lopattananon N., Kettle A.P., Tripathi D., Beck A.J., Duval E., France R.M., Short R.D., Jones F.R. (1999). Interface molecular engineering of carbon-fiber composites. Compos. Part A Appl. Sci. Manuf..

[36] Naito K. (2010). Fracture Behaviour of Continuous Carbon Fibre. Improvement of Resin Impregnation Property and Reliability Evaluation of CFRP (Carbon Fiber Reinforced Plastic).

[37] Galli C. (1988). Radical reactions of arenediazonium ions: An easy entry into the chemistry of the aryl radical. Chem. Rev..

[38] Bahr J.L., Tour J.M. (2001). Highly functionalized carbon nanotubes using in situ generated diazonium compounds. Chem. Mater..

[39] Klemm D., Heublein B., Fink H.-P., Bohn A. (2005). Cellulose: Fascinating biopolymer and sustainable raw material. Angew. Chem. Int. Ed..

[40] Ma L., Meng L., Fan D., He J., Yu J., Qi M., Chen Z., Huang Y. (2014). Interfacial enhancement of carbon fiber composites by generation 1-3 dendritic hexamethylenetetramine functionalization. Appl. Surf. Sci..

[41] Zhang G., Sun S., Yang D., Dodelet J.-P., Sacher E. (2008). The surface analytical characterization of carbon fibers functionalized by H_2_SO_4_/HNO_3_ treatment. Carbon.

[42] Ehlert G.J., Lin Y., Sodano H.A. (2011). Carboxyl functionalization of carbon fibers through a grafting reaction that preserves fiber tensile strength. Carbon.

[43] Meier A.R., Bahureksa W.A., Heien M.L. (2016). Elucidating the structure-function relationship of poly(3,4-theylenedioxythiophene) films to advance electrochemical measurements. J. Phys. Chem. C.

[44] Marsella J.A., Starner W.E. (2000). Acceleration of amine/epoxy reactions with *N*-methyl secondary amines. J. Polym. Sci. Part A Polym. Chem..

[45] Wu Q., Li M., Gu Y., Wang S., Yao L., Zhang Z. (2016). Effect of sizing agent on interfacial adhesion of commercial high strength carbon fiber-reinforced resin composites. Polym. Compos..

[46] Huson M.G., Church J.S., Kafi A.A., Woodhead A.L., Khoo J., Kiran M.S.R.N., Bradby J.E., Fox B.L. (2014). Heterogeneity of carbon fibre. Carbon.

