# Supplementary material for: Lignin as a Functional Green Coating on Carbon Fiber Surface to Improve Interfacial Adhesion in Carbon Fiber Reinforced Polymers
Source: Materials (Basel). 2019 Jan 6;12(1):159. doi: 10.3390/ma12010159 (PMC6337094; doi:10.3390/ma12010159)
Supplement: Supplementary file 1 [file materials-12-00159-s001.pdf]

Supplementary Material for

Lignin as a functional green coating on carbon fiber surface to improve interfacial adhesion in carbon fiber reinforced polymers

László Szabó,[a,*] Sari Imanishi,[a] Fujie Tetsuo,[a] Daisuke Hirose,[a] Hisai Ueda,[b] Takayuki Tsukegi,[b] Kazuaki Ninomiya,[c] Kenji Takahashi[a,*]

[a] Institute of Science and Engineering, Kanazawa University, Kakuma-machi, Kanazawa 920-1192, Japan.

[b] Innovative Composite Center, Kanazawa Institute of Technology, 2-2 Yatsukaho, Hakusan 924-0838, Japan.

[c] Institute for Frontier Science Initiative, Kanazawa University, Kakuma-machi, Kanazawa 920-1192, Japan.

[*] Corresponding authors. E-mail: szabo-laszlo@se.kanazawa-u.ac.jp; ktkenji@staff.kanazawa-u.ac.jp; Tel: +81 76-234-4828.

Table of Contents

1. **^{31}P-NMR results** S3
2. **Microdroplet experiment** S4
3. **Cyclic voltammetry results** S5
4. **XPS analysis** S6
5. **FE-SEM-EDX analysis** S8
6. **SEM images** S11
7. **SEM images of fracture surfaces after the fragmentation test** S12
8. **SEM images of fracture surfaces after the microdroplet test** S13

1. ³¹P-NMR results

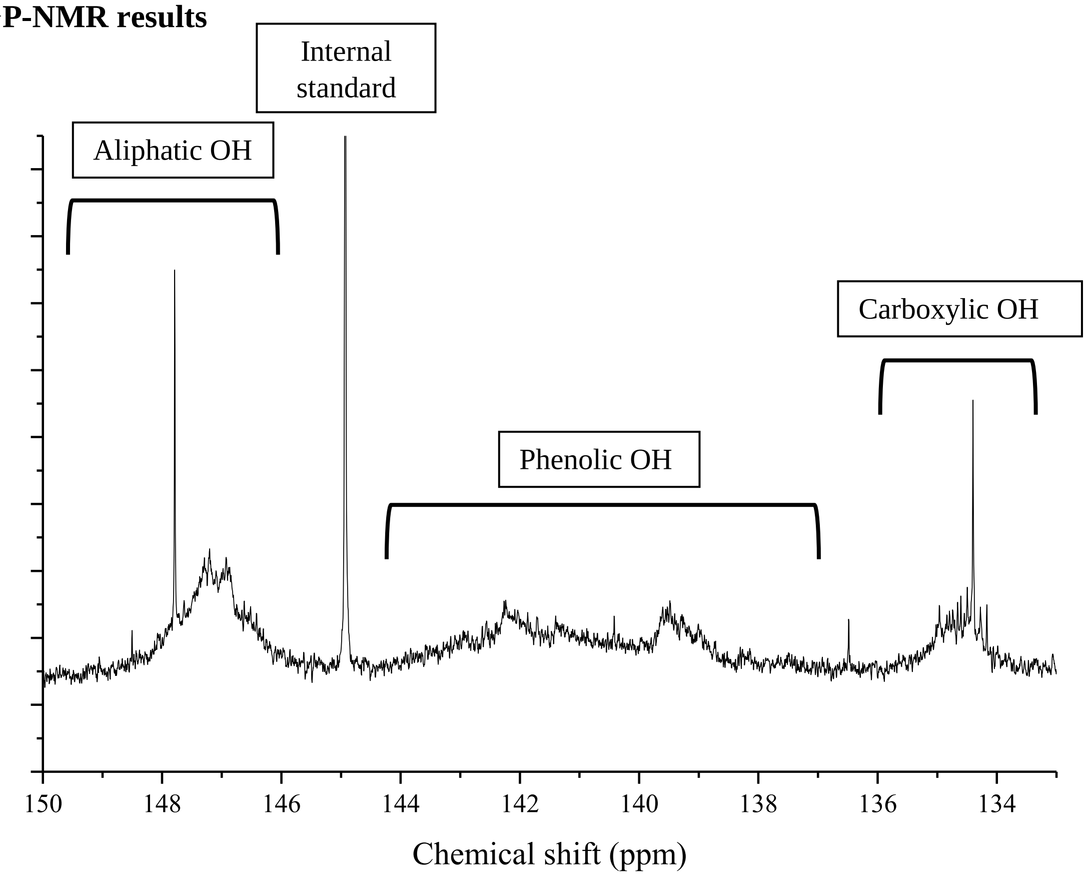

Figure S1. Quantitative ^{31}P-NMR spectrum for determining OH content of kraft lignin sample (as received from Sigma Aldrich) according to Granata and Argyropoulos [1].

2. Microdroplet experiment

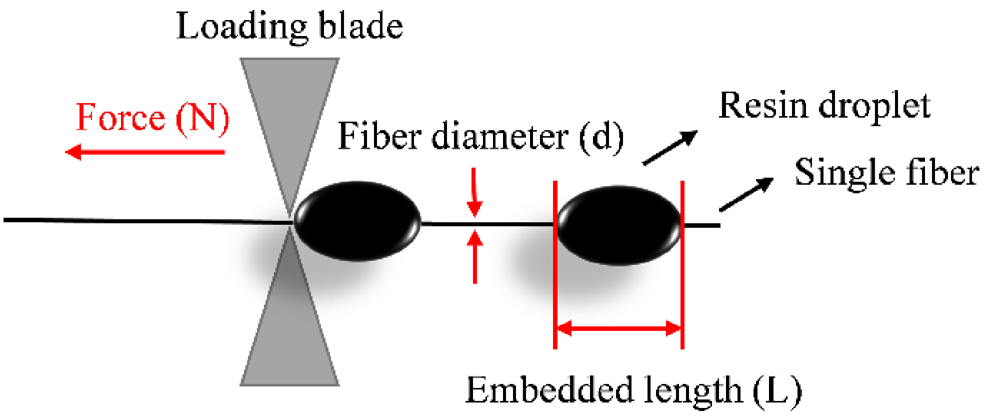

$$\tau_i = \frac{F}{\pi d L}$$

F – maximum load (N)

Figure S2. Microdroplet experimental setup.

3. Cyclic voltammetry results

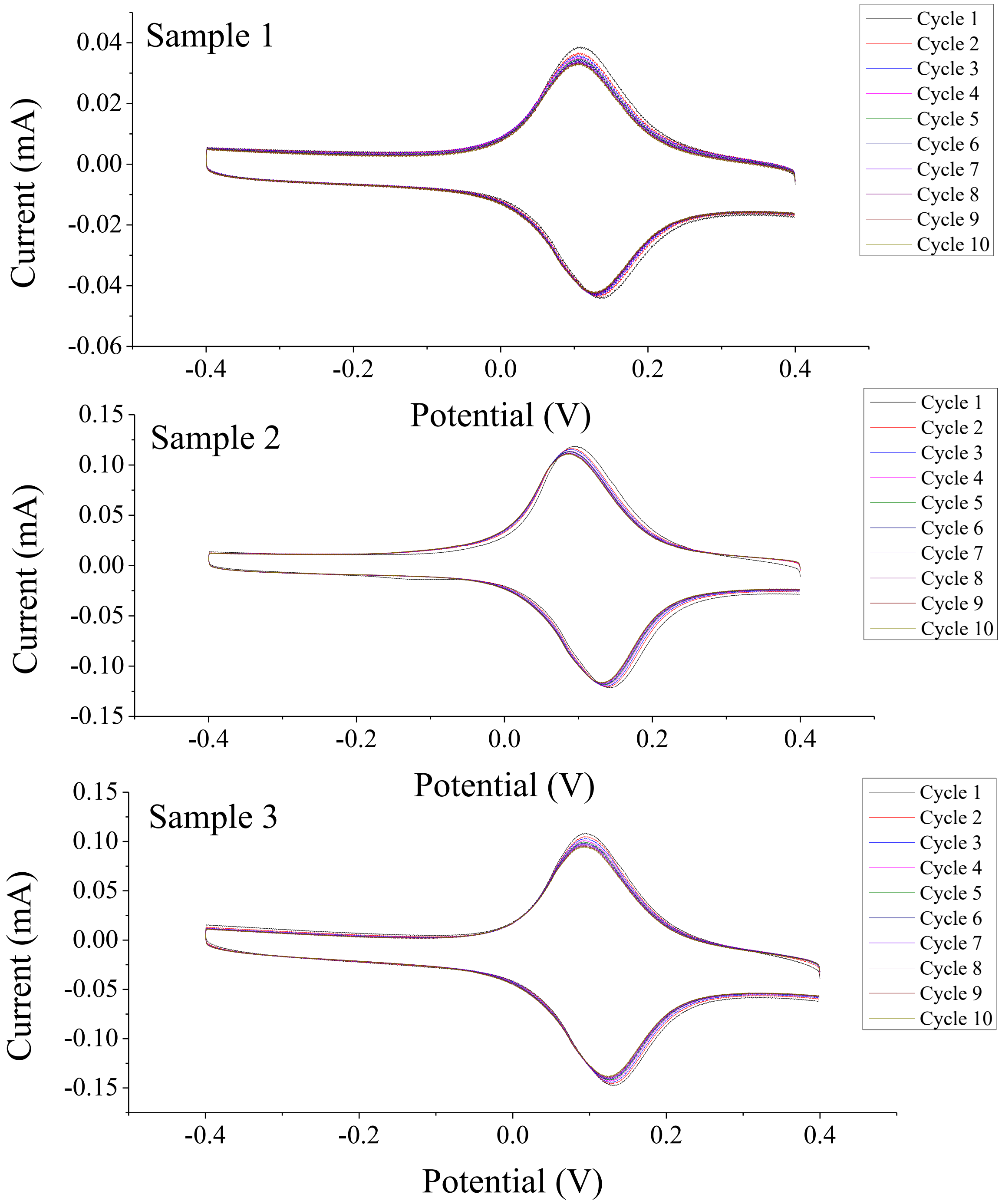

Figure S3. Cyclic voltammetry results to determine grafting density of 4-(aminomethyl)benzene structures modified with the ferrocene/ferrocenium couple (note that the cyclic voltammograms were obtained using different amount of samples).

4. XPS analysis

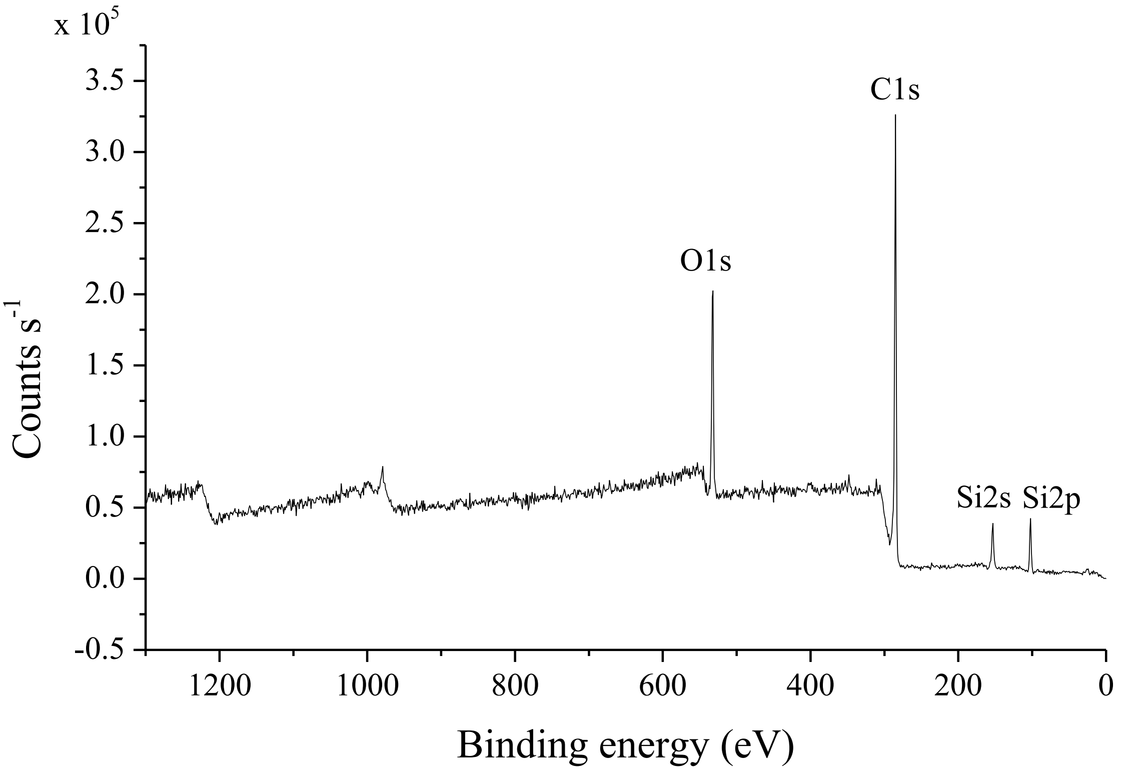

Figure S4. Survey spectrum recorded for carbon fiber sample containing lignin on the surface.

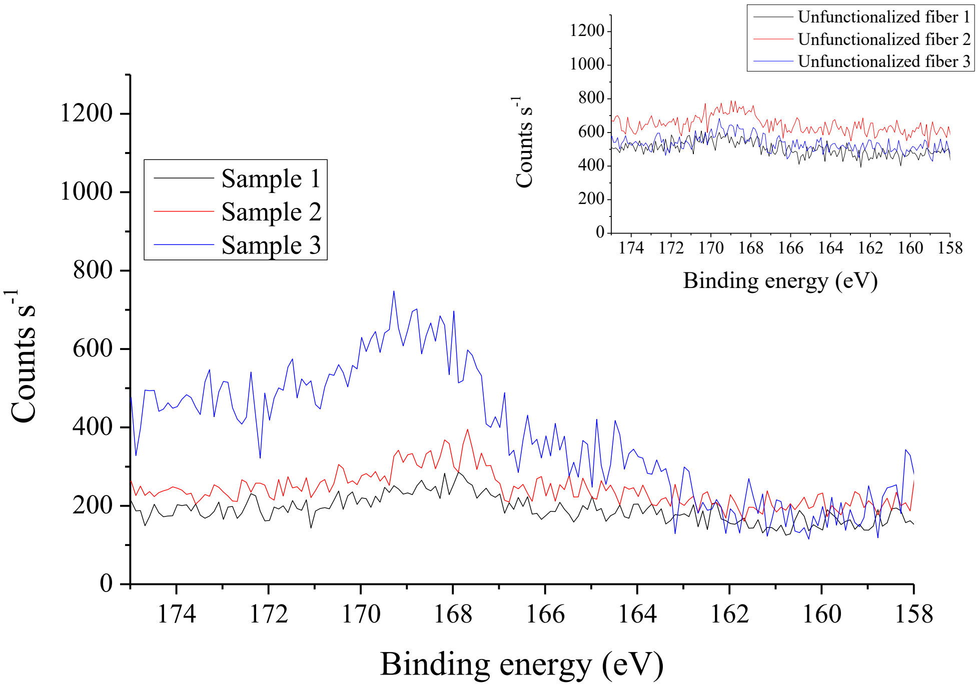

Figure S5. High-resolution S2p spectra of carbon fiber sample containing lignin on the surface with inset displaying the unfunctionalized sample.

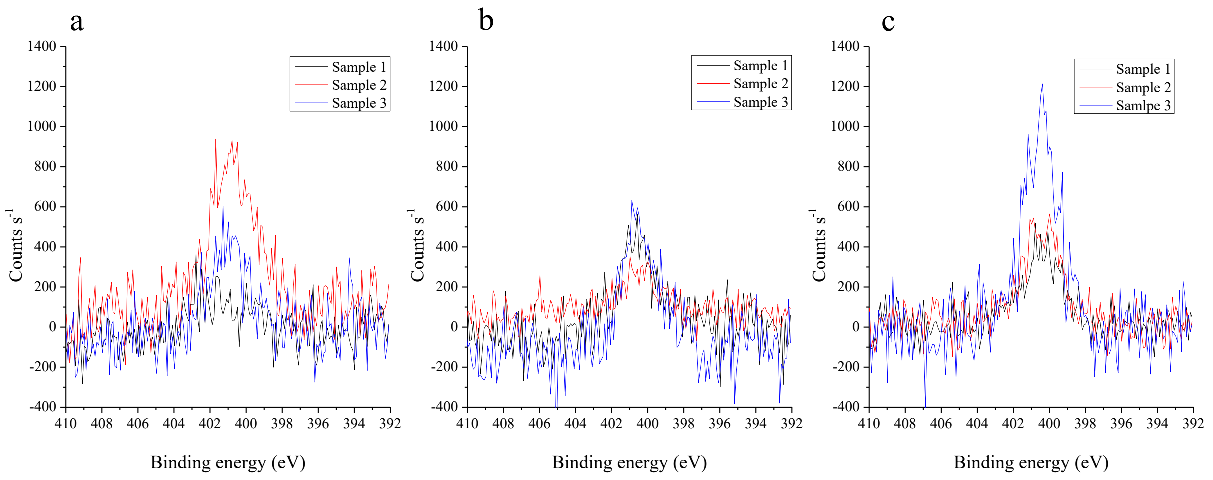

Figure S6. High resolution N1s spectra of (**a**) unfunctionalized carbon fiber, (**b**) 4-(aminomethyl)benzene functionalized carbon fiber and (**c**) lignin functionalized samples.

5. FE-SEM-EDX analysis

Table S1. Elemental composition of the bulk and near-surface layer of carbon fiber (CF) samples.

		C (%)	O (%)	N (%)
**CF - lignin**	**Sample 1**	**91.17**	**5.42**	**2.98**
	**Sample 2**	**92.37**	**6.26**	**1.06**
	**Sample 3**	**90.73**	**3.77**	**5.24**
	**Average**	**91.42**	**5.15**	**3.09**
	**St. deviation**	**0.85**	**1.27**	**2.09**
**Control**	**Sample 1**	**91.09**	**3.88**	**4.71**
	**Sample 2**	**93.15**	**3.00**	**1.85**
	**Sample 3**	**92.95**	**3.19**	**3.53**
	**Average**	**92.40**	**3.36**	**3.40**
	**St. deviation**	**1.14**	**0.46**	**1.44**

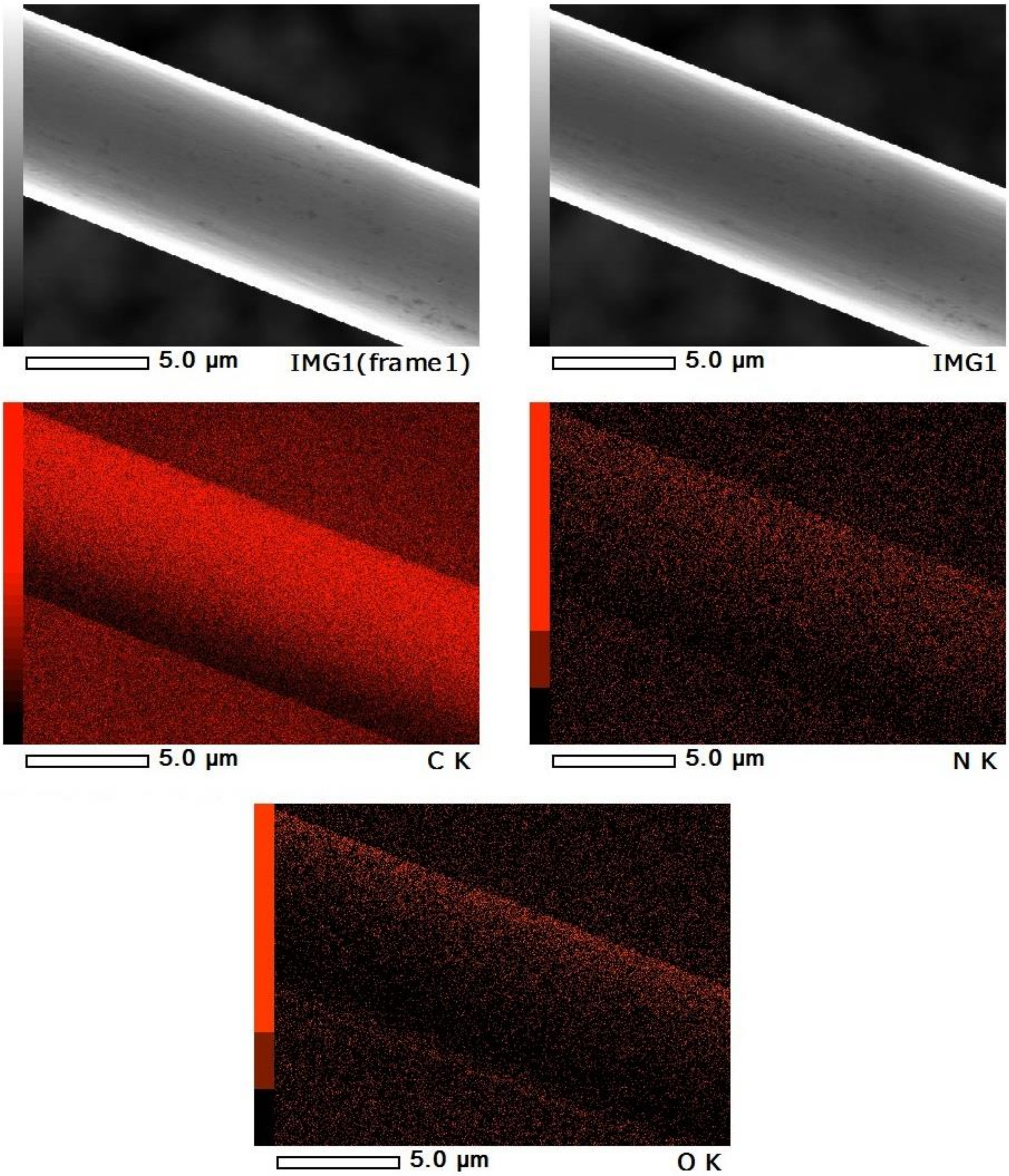

Figure S7. Chemical mapping experiment for carbon fiber sample containing lignin on the surface.

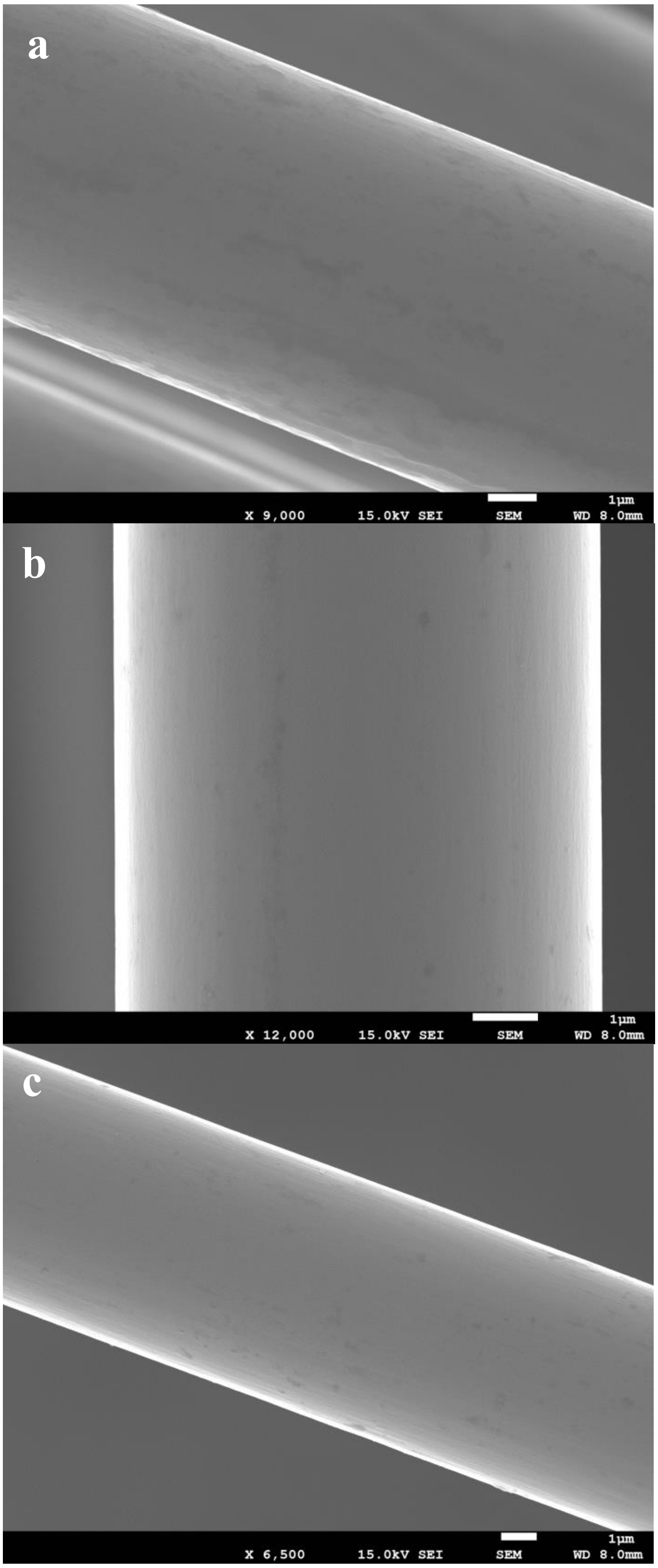

Figure S8. FE-SEM images of (**a**) control carbon fiber (unfunctionalized), (**b**) carbon fiber with sizing agent (as received) and (**c**) carbon fiber containing lignin on the surface.

6. SEM images

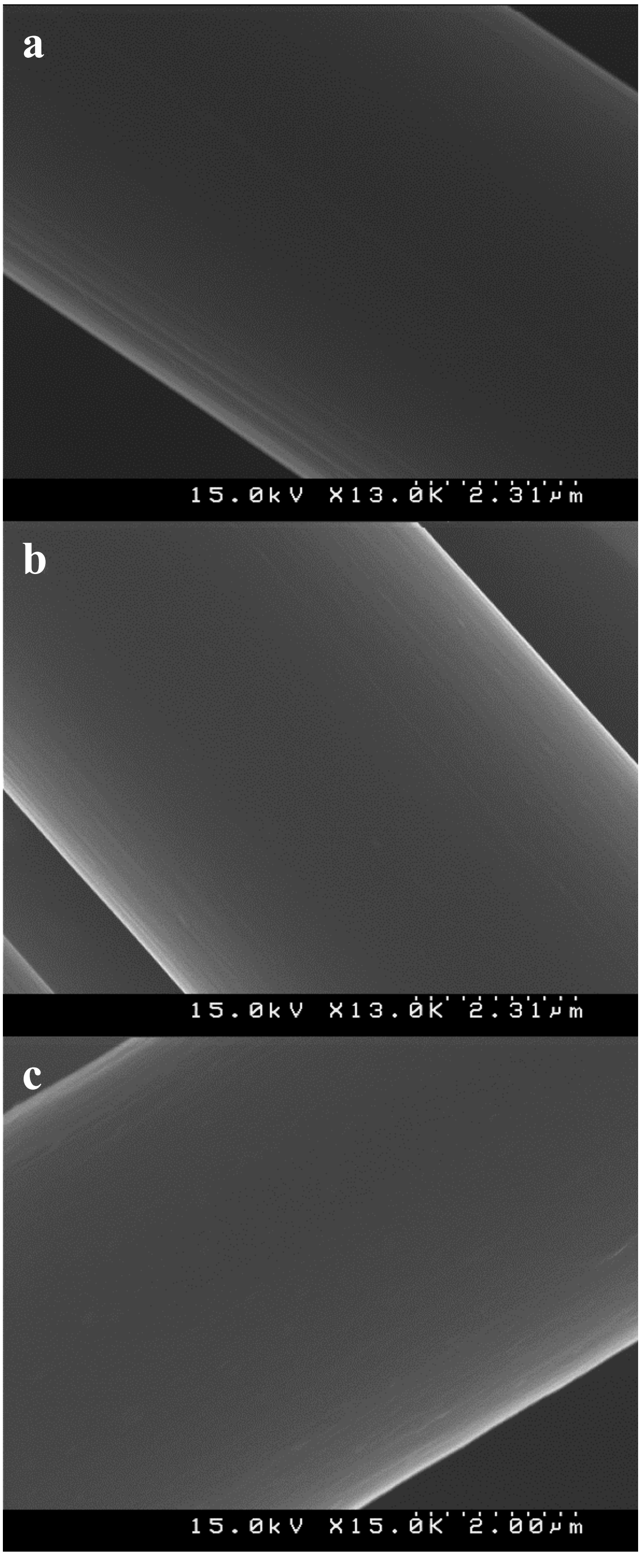

Figure S9. SEM images of (**a**) control carbon fiber (unfunctionalized), (**b**) carbon fiber with sizing agent (as received) and (**c**) carbon fiber containing lignin on the surface.

7. SEM images of fracture surfaces after the fragmentation test

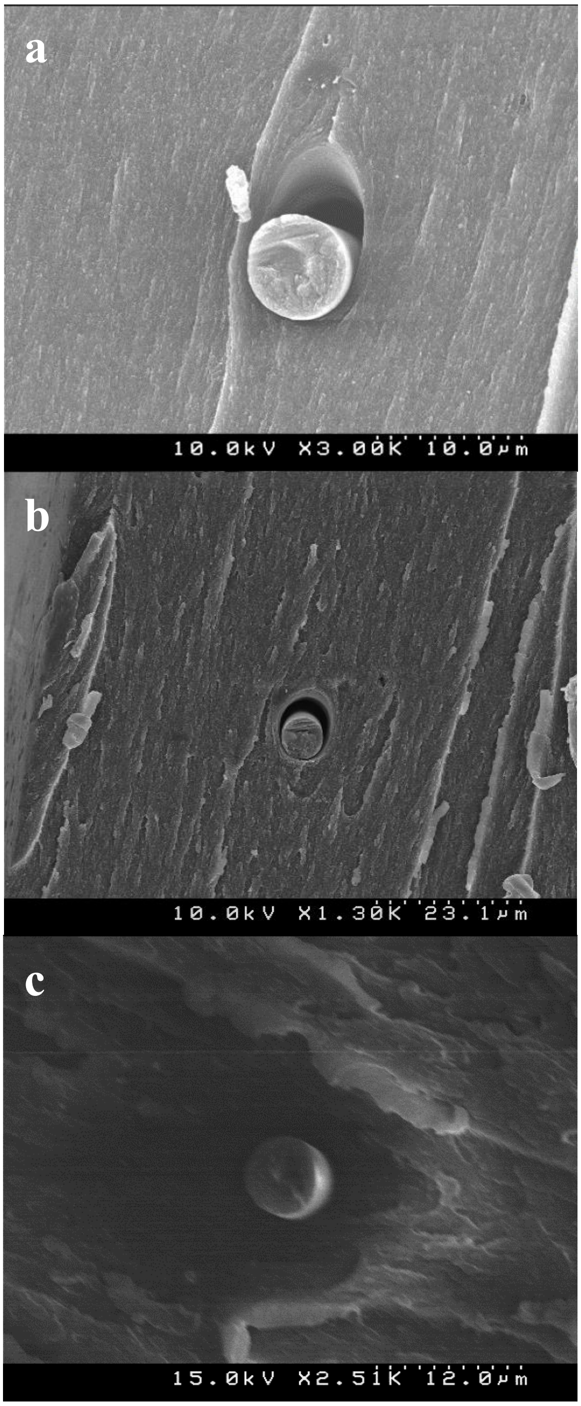

Figure S10. SEM images of fracture surfaces after the fragmentation test for (**a**) unfunctionalized carbon fiber sample, (**b**) carbon fiber functionalized with 4-(aminomethyl)benzene and (**c**) containing lignin on the surface.

8. SEM images of fracture surfaces after the microdroplet test

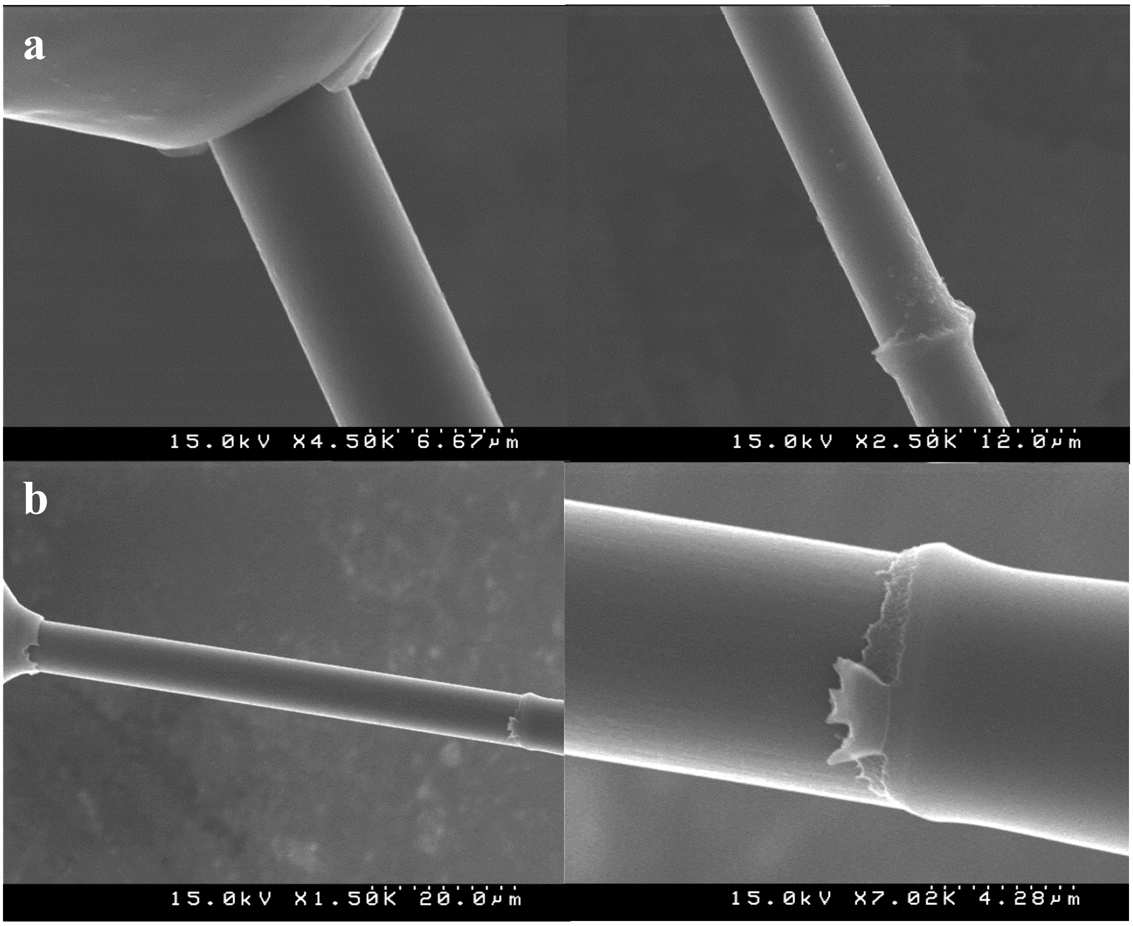

Figure S11. Fracture surfaces recorded after microdroplet test. 4-(Aminomethyl)benzene functionalized carbon fiber samples with (**a**) epoxy and (**b**) cellulose propionate matrix.

References

[1] A Granata, D S Argyropoulos, 2-Chloro-4,4,5,5-tetramethyl-1,3,2-dioxaphospholane, a reagent for the accurate determination of the uncondensed and condensed phenolic moieties in lignins, J Agric Food Chem, 1995, 43, 1538-1544.
